# Home spirometry in patients with idiopathic pulmonary fibrosis: data from the INMARK trial

**DOI:** 10.1183/13993003.01518-2020

**Published:** 2021-07-08

**Authors:** Imre Noth, Vincent Cottin, Nazia Chaudhuri, Tamera J. Corte, Kerri A. Johannson, Marlies Wijsenbeek, Stephane Jouneau, Andreas Michael, Manuel Quaresma, Klaus B. Rohr, Anne-Marie Russell, Susanne Stowasser, Toby M. Maher

**Affiliations:** 1 Division of Pulmonary and Critical Care Medicine, University of Virginia, Charlottesville, VA, USA; 2 National Reference Centre for Rare Pulmonary Diseases, Louis Pradel Hospital, Hospices Civils de Lyon, Claude Bernard University Lyon 1, Lyon, France; 3 North West Interstitial Lung Disease Unit, Manchester University NHS Foundation Trust, Manchester, UK; 4 Royal Prince Alfred Hospital, Camperdown, Australia; 5 University of Sydney, Sydney, Australia; 6 Medicine and Community Health Sciences, University of Calgary, Calgary, AB, Canada; 7 Dept of Respiratory Medicine, Erasmus MC, University Medical Center, Rotterdam, The Netherlands; 8 Hôpital Pontchaillou – CHU de Rennes, IRSET UMR 1085, Université de Rennes 1, Rennes, France; 9 Syneos Health, Farnborough, UK; 10 Boehringer Ingelheim International GmbH, Ingelheim am Rhein, Germany; 11 College of Medicine and Health, University of Exeter, Exeter, UK; 12 National Heart and Lung Institute, Imperial College London, London, UK; 13 National Institute for Health Research Clinical Research Facility, Royal Brompton Hospital, London, UK; 14 Keck School of Medicine, University of Southern California, Los Angeles, CA, USA

## Abstract

**Background:**

Data from the INMARK trial were used to investigate the feasibility and validity of home spirometry as a measure of lung function decline in patients with idiopathic pulmonary fibrosis (IPF).

**Methods:**

Subjects with IPF and preserved forced vital capacity (FVC) were randomised to receive nintedanib or placebo for 12 weeks followed by open-label nintedanib for 40 weeks. Clinic spirometry was conducted at baseline and weeks 4, 8, 12, 16, 20, 24, 36 and 52. Subjects were asked to perform home spirometry at least once a week and ideally daily. Correlations between home- and clinic-measured FVC and rates of change in FVC were assessed using Pearson correlation coefficients.

**Results:**

In total, 346 subjects were treated. Mean adherence to weekly home spirometry decreased over time but remained above 75% in every 4-week period. Over 52 weeks, mean adherence was 86%. Variability in change from baseline in FVC was greater when measured by home rather than clinic spirometry. Strong correlations were observed between home- and clinic-measured FVC at all time-points (r=0.72–0.84), but correlations between home- and clinic-measured rates of change in FVC were weak (r=0.26 for rate of decline in FVC over 52 weeks).

**Conclusion:**

Home spirometry was a feasible and valid measure of lung function in patients with IPF and preserved FVC, but estimates of the rate of FVC decline obtained using home spirometry were poorly correlated with those based on clinic spirometry.

## Abstract 2

**In a 52-week study in 346 subjects with idiopathic pulmonary fibrosis, mean adherence to weekly home spirometry was 86%. Estimates of the rate of decline in forced vital capacity obtained using home and clinic spirometry were poorly correlated.** https://bit.ly/2WjIQ4b

## Introduction

Idiopathic pulmonary fibrosis (IPF) is a chronic fibrosing interstitial lung disease (ILD) characterised by decline in lung function [1]. Although IPF is always progressive, the rate and pattern of forced vital capacity (FVC) decline are variable among individuals [1–3]. Lung function has traditionally been measured periodically in a clinic-based setting, supervised by trained clinicians, but measurements obtained at home using a hand-held device have been shown to correlate well with clinic-based measurements over a 3–12-month period [4–8]. Home spirometry may offer advantages over clinic spirometry by increasing convenience for patients and providing more frequent measurements of lung function, enabling earlier detection of disease progression or acute exacerbations [4, 6, 9]. More frequent assessment of lung function *via* home spirometry might also provide improved analytical sensitivity, reducing the sample size required to power clinical trials [6]. However, in a recent trial conducted in subjects with unclassifiable ILD, the pre-specified analysis model could not be applied to the home spirometry measurements, in part due to issues with the reliability of the measurements [10]. More data are needed on the utility of home spirometry in the monitoring of lung function both in clinical trials and clinical practice.

In the INMARK trial in subjects with IPF and preserved lung function, lung function was assessed using both home and clinic spirometry over 52 weeks [11]. We used data from the INMARK trial to assess the feasibility and validity of home spirometry as a measure of lung function decline in subjects with IPF.

## Methods

### Study design and subjects

The primary objective of the INMARK trial was to investigate the effects of nintedanib on circulating biomarkers. The trial design has been described [11]. Briefly, subjects who had been diagnosed with IPF in the previous 3 years and had FVC ≥80% predicted were randomised 1:2 to receive nintedanib 150 mg twice daily or placebo for 12 weeks, followed by an open-label period in which all subjects received nintedanib 150 mg twice daily for 40 weeks [11]. Home spirometry devices (SpiroPro; ERT, Philadelphia, PA, USA) and instructions were given to subjects at screening. To be eligible for the trial, subjects were required to perform one or more home spirometry readings between screening and randomisation (a period of ≤28 days). The last measurement taken prior to the first intake of nintedanib or placebo was used as the baseline measurement.

### Home and clinic spirometry

Subjects were asked to perform home spirometry (with three or more efforts) at least once a week, and ideally daily, throughout the trial. The highest value of the three or more efforts was recorded as the measurement. Subjects were asked to perform home spirometry in the morning, preferably between 08:00 and 11:00 h. An acoustic alarm on the device was activated daily at 09:00 and 09:30 h if the subject had not completed one or more efforts. For every measurement, the device showed the subject their highest value for FVC % predicted (calculated according to Quanjer *et al.* [12]) and informed them if they had experienced a relative decline in FVC ≥10% predicted from baseline; in this instance, subjects were advised to call their doctor. At each visit, subjects were re-trained on how to perform home spirometry if their adherence to weekly home spirometry since the last visit was <80% or as deemed necessary by the site. Adherence to weekly home spirometry was calculated as the number of weeks that a subject provided one or more measurements divided by the number of weeks that they were followed in the trial. Thus, 100% adherence was defined as provision of one or more measurements per week for all the weeks that the subject was in the trial.

Clinic spirometry was conducted at baseline and weeks 4, 8, 12, 16, 20, 24, 36 and 52. Clinic spirometry was centrally reviewed, and ongoing feedback and training were provided to the sites.

### Analyses

Correlations between the following assessments at every time-point were assessed using the Pearson correlation coefficient (r): home and clinic measurements of FVC (mL), forced expiratory volume in 1 s (FEV_1_) (mL) and forced expiratory volume in 6 s (FEV_6_) (mL), home and clinic measurements of changes from baseline in FVC (mL), FEV_1_ (mL) and FEV_6_ (mL), and home and clinic measurements of rates of decline in FVC (mL), FEV_1_ (mL) and FEV_6_ (mL). In the analysis of correlations, the home measurement performed closest to the clinic visit was used (but the home spirometry device did not capture a measurement on the same day as a clinic visit).

The annual rates of decline in FVC and FEV_6_ were assessed using random coefficient piecewise regression with fixed effects for sex, age and height, and random effects of patient-specific intercept, time and a piecewise knot at week 12. Acute exacerbations, defined as in the INPULSIS trials [13], were reported by investigators using a tick box on the case report form and were not adjudicated. In subjects who had an investigator-reported acute exacerbation, all available home and clinic measurements of FVC (mL) before and after the acute exacerbation were plotted. Analyses were conducted using SAS (SAS Institute, Cary, NC, USA). Analyses were descriptive and exploratory.

## Results

A total of 346 subjects were treated in the INMARK trial (116 randomised to nintedanib and 230 randomised to placebo). At baseline, mean±sd FVC was 3305±1060 mL and 99.6±23.8% predicted based on home spirometry and 3241±812 mL and 97.5±13.5% predicted based on clinic spirometry. In total, 83.5% of the subjects who were randomised completed 52 weeks of treatment.

### Annual rate of decline in FVC and FEV_6_

In subjects treated with nintedanib for 52 weeks, the adjusted mean±se home- and clinic-measured rates of FVC decline were −127.2±76.3 and −88.8±23.9 mL·year^−1^, respectively, and the adjusted mean±se home- and clinic-measured rates of FEV_6_ decline were −112.6±69.5 and −90.5±22.3 mL·year^−1^, respectively. In subjects treated with placebo for 12 weeks followed by nintedanib for 40 weeks, the adjusted mean±se home- and clinic-measured rates of FVC decline were −111.8±54.7 and −104.1±17.0 mL·year^−1^, respectively, and the adjusted mean±se home- and clinic-measured rates of FEV_6_ decline were −131.8±49.9 and −103.9±15.9 mL·year^−1^, respectively.

### Adherence to home spirometry

**FIGURE 1:**
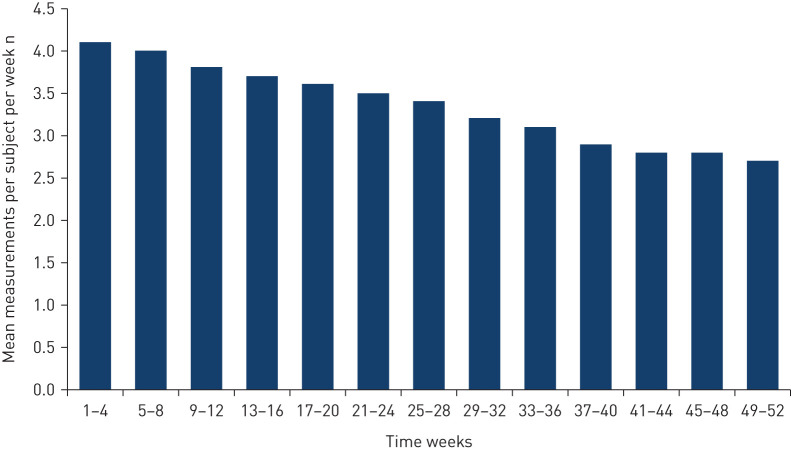
Mean number of home spirometry measurements per subject per week. Analysis based on the total number of home spirometry measurements collected and the number of subjects who were still followed in the trial within the time period.

Over 52 weeks, the mean±sd number of home spirometry measurements per subject was 165±115 (table 1). The mean±sd number of measurements per subject per week was 3.4±2.6 and the median was 3.0. The mean number of measurements per subject per week decreased over the trial but remained above 2.5 in every 4-week period (figure 1).

**TABLE 1:** Home spirometry measurements per subject over 52 weeks

	**Nintedanib**	**Placebo/nintedanib^#^**	**All subjects**
**Subjects**	116	230	346
**Home spirometry measurements**			
Mean±sd	157±106	170±119	165±115
Minimum	3	3	3
Median	125	136	132
Maximum	362	633	633

Data are presented as n, unless otherwise stated. ^#^: subjects received placebo (blinded) for 12 weeks followed by open-label nintedanib for 40 weeks.

**FIGURE 2:**
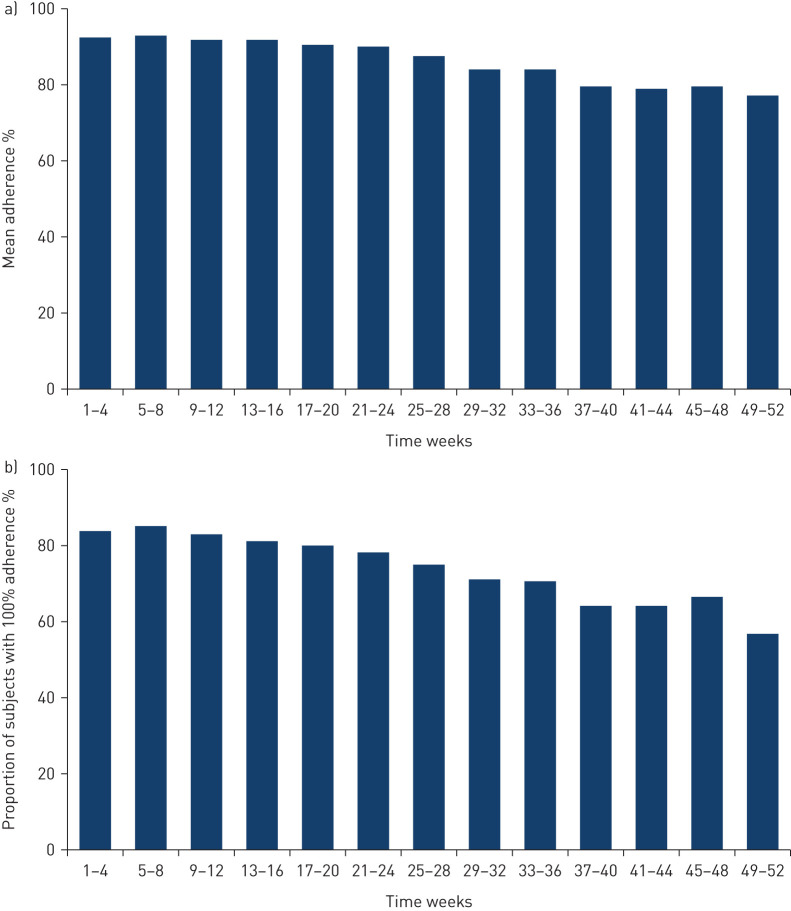
a) Mean adherence to weekly home spirometry. Adherence to weekly home spirometry was calculated as the number of weeks that a subject provided at least one measurement divided by the number of weeks that they were followed in the trial. b) Proportion of subjects with 100% adherence to weekly home spirometry. 100% adherence was defined as provision of at least one measurement per week for all the weeks that the subject was in the trial. The total number of subjects who were still followed in the trial within the time period was used as the denominator.

Over 52 weeks, mean and median adherence to weekly home spirometry were 86% and 96%. Mean adherence to weekly home spirometry decreased over the trial but remained above 75% in every 4-week period (figure 2a). The proportion of subjects with 100% adherence decreased over the trial but remained above 50% in every 4-week period (figure 2b and supplementary figure S1). Over 52 weeks, 31% of subjects had 100% adherence to weekly home spirometry.

Subjects who had 100% adherence to weekly home spirometry (n=108) had slightly higher mean FVC and diffusing capacity of the lung for carbon monoxide at baseline than subjects who had <100% adherence (n=238) (supplementary table S1). Permanent discontinuation of trial medication was less common among subjects with 100% *versus* <100% adherence to weekly home spirometry (4.6% *versus* 21.8%).

### Timing of home spirometry measurements

Over 52 weeks, 45.7% of subjects provided only one measurement on any day on which they provided a measurement. Most subjects took some of their measurements in the morning (defined as between 05:00 and 12:00 h) and some in the afternoon/evening (defined as between 12:00 and 05:00 h) (supplementary figures S2 and S3). The mean±sd FVC at baseline was similar between measurements taken in the morning and the afternoon/evening (3379±1062 and 3344±1277 mL, respectively). The mean FVC over time was variable, with greater variability in the measurements taken in the afternoon/evening than in the morning (supplementary figure S4).

### Correlations between FVC and FEV_6_ measured using home and clinic spirometry

**FIGURE 3:**
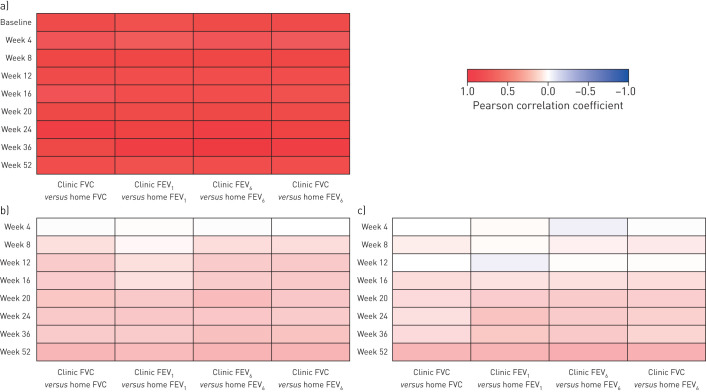
“Heatmaps” depicting correlations between a) lung function variables measured at home and in clinic at different time-points (r≥0.5 for all correlations), b) changes from baseline in lung function variables measured at home and in clinic at different time-points, and c) rates of decline in lung function variables measured at home and in clinic at different time-points. FVC: forced vital capacity; FEV_1_: forced expiratory volume in 1 s; FEV_6_: forced expiratory volume in 6 s. Darker shades of red or blue indicate stronger positive or negative associations, respectively.

Correlations between FVC and FEV_6_, and changes in FVC and FEV_6_, measured using home and clinic spirometry are presented in figures 3a–c. Strong correlations were observed between home and clinic measurements of FVC (r=0.72–0.84), home and clinic measurements of FEV_6_ (r=0.71–0.85), and clinic measurements of FVC and home measurements of FEV_6_ (r=0.71–0.84) at all individual time-points (figure 3a). Correlations between home and clinic measurements of FVC were weaker in subjects who provided more than three *versus* three or less home spirometry measurements per week (r=0.63–0.75 *versus* r=0.78–0.94).

**FIGURE 4:**
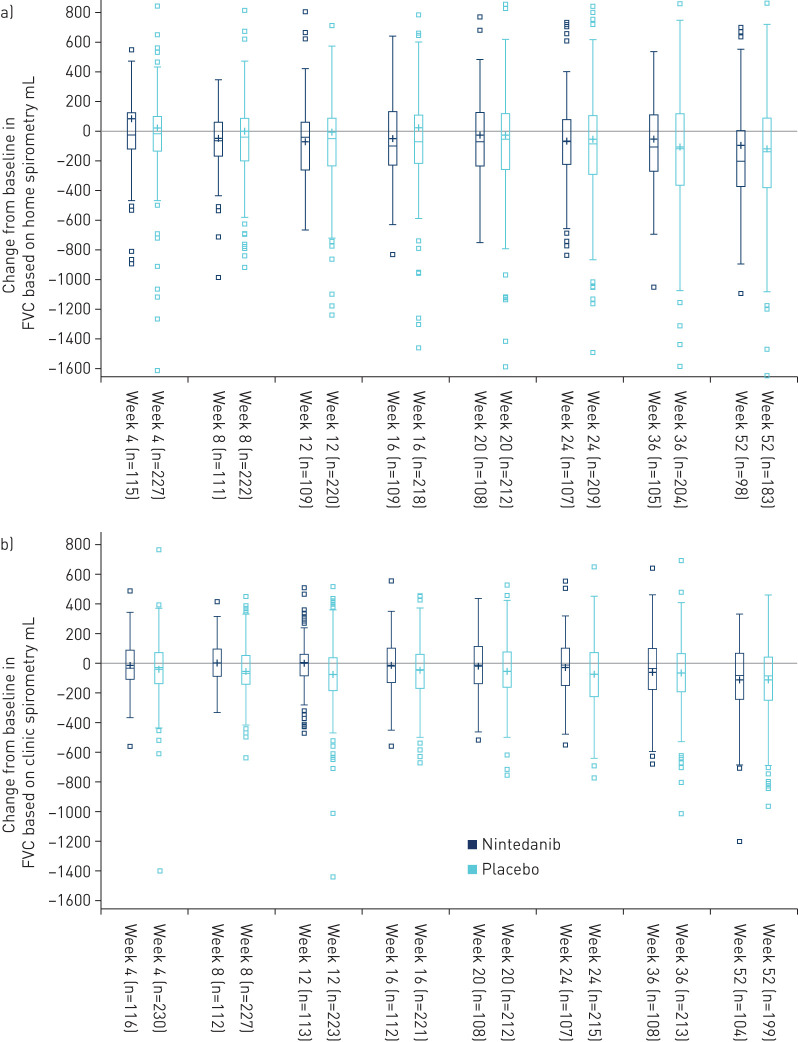
Changes from baseline in forced vital capacity (FVC) based on a) home spirometry and b) clinic spirometry. Boxes indicate median and interquartile range (IQR); “plus” symbols (+) indicate mean. Whiskers indicate 1.5 IQR. Outliers are shown as squares.

The variability in change from baseline in FVC was greater when measured using home spirometry than clinic spirometry (figure 4). Correlations between home- and clinic-measured changes from baseline in FVC were weak but increased over 52 weeks (r= –0.01 at week 4 and r=0.25 at week 52). Similar correlations were observed for FEV_6_ (r= –0.01 at week 4 and r=0.27 at week 52) (figure 3b).

Correlations between home- and clinic-measured rates of change in FVC were weak but increased over 52 weeks (r=0.00 and r=0.26 for rates of decline in FVC over 4 and 52 weeks, respectively). Similar correlations were observed for rates of change in FEV_6_ (r= –0.05 and r=0.29 over 4 and 52 weeks, respectively) (figure 3c).

### Home and clinic spirometry in subjects who had an acute exacerbation

One subject in the nintedanib group had an acute exacerbation during the double-blind period and seven subjects who initially received placebo had an acute exacerbation during the nintedanib open-label period. Home and clinic measurements of FVC before and after these acute exacerbations are presented in supplementary figure S5.

## Discussion

In the INMARK trial conducted in subjects with IPF and preserved lung function, adherence to weekly home spirometry over 52 weeks was above 75% in every 4-week period, but decreased over time. Over 52 weeks, 31% of subjects adhered to the request to provide at least one measurement per week for all the weeks they were in the trial. A proportion of subjects provided more measurements than the minimum requested, with an average of three measurements per subject per week. These findings are consistent with previous studies in patients with ILDs that have demonstrated high adherence to daily or weekly home spirometry, but with high variability among individuals and a reduction in the number of measurements provided over time [6, 8, 14]. Previous work suggests that patients with IPF find home spirometers easy to use and not burdensome, and that patients like to see their FVC results to feel more in control of their disease [6, 15, 16]. A study of 30 subjects found that only four were unable to use the home spirometry device [6].

Within-subject variability in FVC measurements taken day-to-day or week-to-week has been observed in healthy individuals [17] as well as in subjects with IPF [4, 6]. The literature is inconsistent with respect to diurnal variations in FVC; several studies have found FVC to be generally higher in the morning than in the afternoon [18–20], but this has not been observed in all studies [21]. In the INMARK trial, subjects were asked to perform spirometry in the morning, but fewer than a third of subjects adhered to this request. The mean of FVC measurements taken in the morning was almost the same as the mean of measurements taken in the afternoon/evening, but, consistent with a previous study [20], variability appeared to be greater in measurements taken in the afternoon/evening than in the morning.

Consistent with previous studies [4, 6–8, 15, 16], we found that home and clinic measurements of FVC at individual visits were strongly correlated. However, there was only a weak correlation between home- and clinic-based measurements of changes in FVC. This appeared to be largely due to variability in changes in FVC measured using home spirometry, which was much greater than the variability observed using clinic spirometry. Errors in measurements taken at different time-points accumulate, such that measurement error has a greater impact on assessments of changes in FVC over time, which are based on several measurements, than on measurements taken at single time-points. While it may be hypothesised that more frequent home spirometry (*i.e.* more data points) might provide a more accurate estimate of lung function, in our study correlations between home and clinic measurements of FVC were weaker in subjects who provided more spirometry measurements per week, likely due to a greater number of outliers. This was observed despite the home spirometry device selecting the highest of three readings for every measurement. Improving the accuracy of home-based spirometry might overcome this problem. To date, no head-to-head comparisons of different spirometers have been undertaken to assess whether particular devices are easier to use correctly and associated with lower measurement error. The correlations between home- and clinic-measured FVC at baseline and at week 52 were the same, suggesting that there was no increase in the reliability of home spirometry during the trial. It has been proposed that the abbreviated FEV_6_ manoeuvre may be easier for patients to perform than measurement of FVC and so improve reproducibility among unsupervised subjects [22]. However, in our analyses the correlations between home and clinic measurements of FVC were almost the same as the correlations between home and clinic measurements of FEV_6_.

It has been postulated that more frequent measurement of FVC at home might enable earlier detection of an acute exacerbation. In a pilot study performed in 10 subjects, a decline in FVC based on daily home spirometry was observed 2 days before symptoms of a respiratory tract infection [15]. We were unable to perform a robust investigation into whether acute exacerbations could be detected earlier using more frequent home spirometry using our data given the small number of acute exacerbations reported in this population with very well preserved FVC at baseline and the low frequency of home spirometry measurements around the time of acute exacerbations.

Although not observed in the INMARK trial, technical issues with home spirometry devices and analytical issues arising from missing data have affected the analysis of home spirometry data from clinical studies in patients with ILDs [23], including trials of potential new therapies [10, 24]. More data are needed to inform strategies to ensure the quality of readings and reduce the variability of measurements obtained using home spirometry by better educating and motivating patients on the use of spirometry devices. It might be possible to reduce the amount of missing data and the variability of home spirometry measurements *via* local support from nurses or other healthcare professionals, or *via* closer or more regular examination of data so that any issues can be addressed promptly with the patient. A recent 24-week study in 90 patients with IPF that investigated the utility of a home monitoring programme integrating daily home spirometry, patient-reported outcomes, adverse event reporting, an information library and electronic consultations found home spirometry to be a reliable and accurate way of monitoring FVC [16]. Median adherence to daily home spirometry over 24 weeks was high (97%), and correlations between home- and hospital-based measurements of FVC were strong at all time-points. Unlike in the INMARK trial, in this study the correlation between the rates of change in home- and hospital-based measurements of FVC was moderately strong (r=0.58) [16].

Strengths of our analyses include the prospective multicentre design and the high frequency and volume of clinic and home spirometry measurements collected. Our findings also have limitations, including selection bias in the subjects who participated in the study, all of whom had preserved lung function at baseline, had shown a degree of adherence to home spirometry before entering the study and had chosen to enter a study that required home spirometry. We were unable to investigate whether comorbid asthma or chronic obstructive pulmonary disease had an impact on spirometry as so few patients in our study had these comorbidities. Our study did not collect data on subjects’ opinions (positive or negative) of home spirometry or on the reasons behind adherence/nonadherence to home spirometry.

In conclusion, in patients with IPF and preserved lung function, adherence to weekly home spirometry decreased over 52 weeks but remained high. Strong correlations were observed between FVC measurements obtained at home and in clinic at individual time-points, but correlations between changes in FVC measurements over time estimated using home and clinic spirometry were weak, mainly due to variability in the measurements obtained using home spirometry. At a group level, the rate of decline in FVC over 52 weeks was similar when measured using home or clinic spirometry. More data are needed on the utility of home spirometry as a means of measuring disease progression in patients with IPF in clinical trials and clinical practice.

## Supplementary material

10.1183/13993003.01518-2020.Supp1 **Please note:** supplementary material is not edited by the Editorial Office, and is uploaded as it has been supplied by the author.
Supplementary material ERJ-01518-2020.SUPPLEMENT

## Shareable PDF

10.1183/13993003.01518-2020.Shareable1 This one-page PDF can be shared freely online.
Shareable PDF ERJ-01518-2020.Shareable
