# Supplementary material for: Home spirometry in patients with idiopathic pulmonary fibrosis: data from the INMARK trial
Source: Eur Respir J. 2021 Jul 8;58(1):2001518. doi: 10.1183/13993003.01518-2020 (PMC8264778; doi:10.1183/13993003.01518-2020)
Supplement: Supplementary file 1 [file ERJ-01518-2020.SUPPLEMENT.pdf]

Supplementary appendix

Table S1. Baseline characteristics of subjects with 100% and <100% adherence to weekly home spirometry

	100% adherence to weekly home spirometry (n=108)	<100% adherence to weekly home spirometry (n=238)
Age, years, mean (SD)	69.9 (7.3)	70.5 (7.4)
Male, n (%)	84 (77.8)	178 (74.8)
Race, n (%)		
White	59 (54.6)	155 (65.1)
Asian	35 (32.4)	68 (28.6)
Missing	14 (13.0)	15 (6.3)
Weight, kg, mean (SD)	79.2 (17.0)	76.9 (15.4)
Body mass index, kg/m^2, mean (SD)	27.8 (4.5)	27.2 (4.1)
Former or current smoker, n (%)	81 (75.0)	171 (71.8)
FVC, mL, mean (SD)	3333 (785)	3200 (822)
FVC, % predicted, mean (SD)	99.2 (15.0)	96.8 (12.8)
DLco*, % predicted, mean (SD)	69.8 (21.2)	61.3 (18.6)

100% adherence was defined as the provision of ≥1 measurement per week for all the weeks the subject was in the trial. *Corrected for haemoglobin.

Figure S1. Proportion of subjects with 100% adherence to home spirometry by treatment group

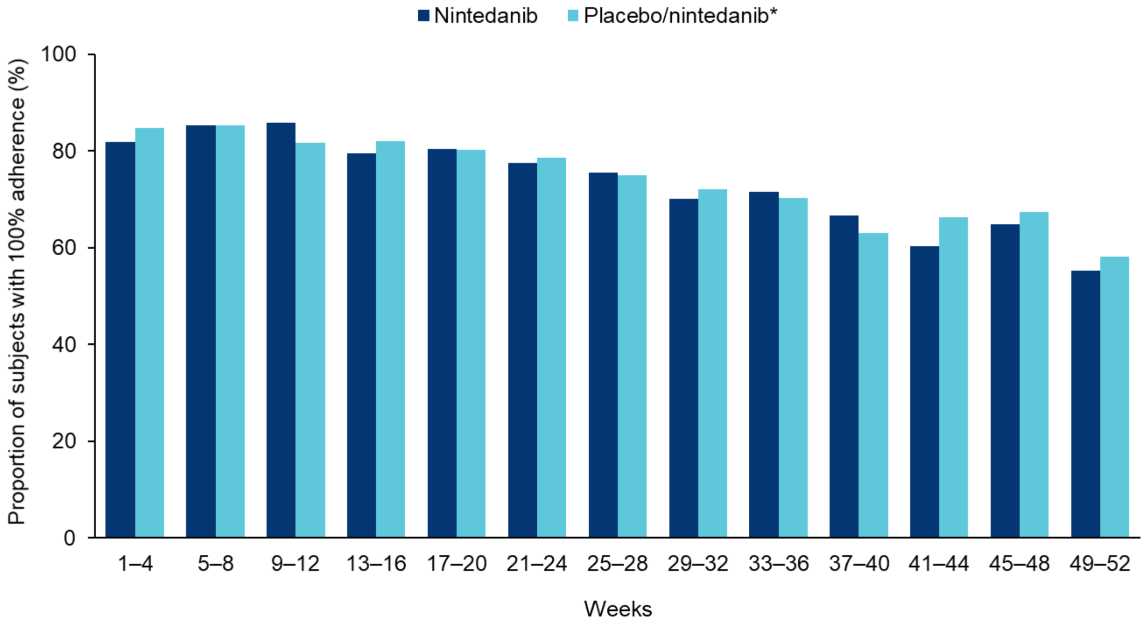

100% adherence was defined as provision of ≥1 measurement per week for all the weeks that the subject was in the trial.
The total number of subjects who were still followed in the trial within the time period was used as the denominator.
*Subjects received placebo (blinded) for 12 weeks followed by open-label nintedanib for 40 weeks.

Figure S2. Timing of home spirometry among subjects who provided only one measurement in a given day

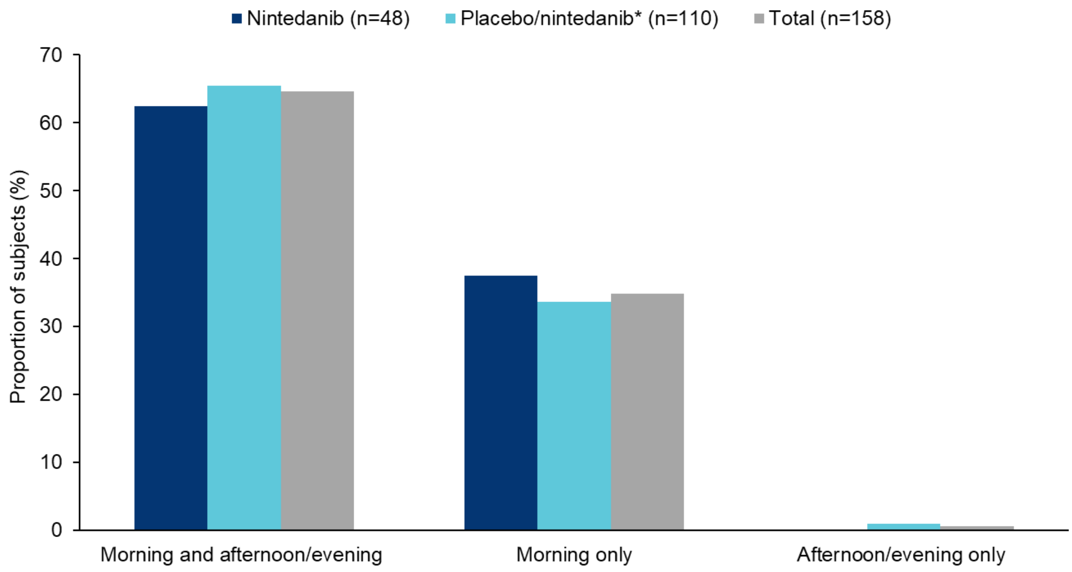

Morning measurements were taken between 05:00 and 12:00 (inclusive). Afternoon/evening measurements were taken after 12:00 and before 05:00.
*Subjects received placebo (blinded) for 12 weeks followed by open-label nintedanib for 40 weeks.

Figure S3. Timing of home spirometry among subjects who provided multiple measurements on at least 1 day

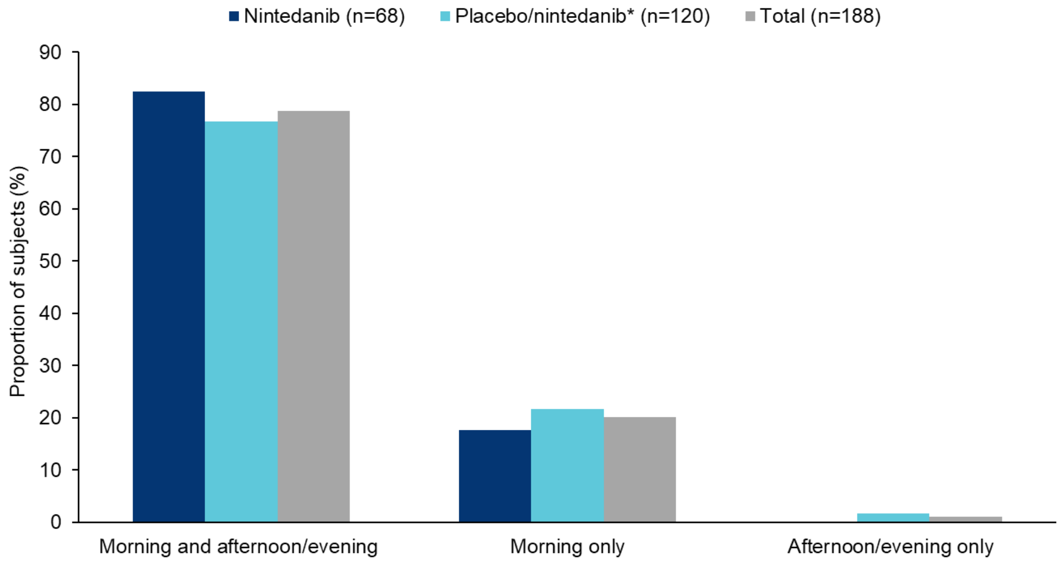

The number of subjects who provided multiple measurements on at least 1 day was used as the denominator.
Morning measurements were taken between 05:00 and 12:00 (inclusive). Afternoon/evening measurements were taken after 12:00 and before 05:00.
*Subjects received placebo (blinded) for 12 weeks followed by open-label nintedanib for 40 weeks.

Figure S4. FVC (mL) based on home spirometry measured in the morning and the afternoon/evening

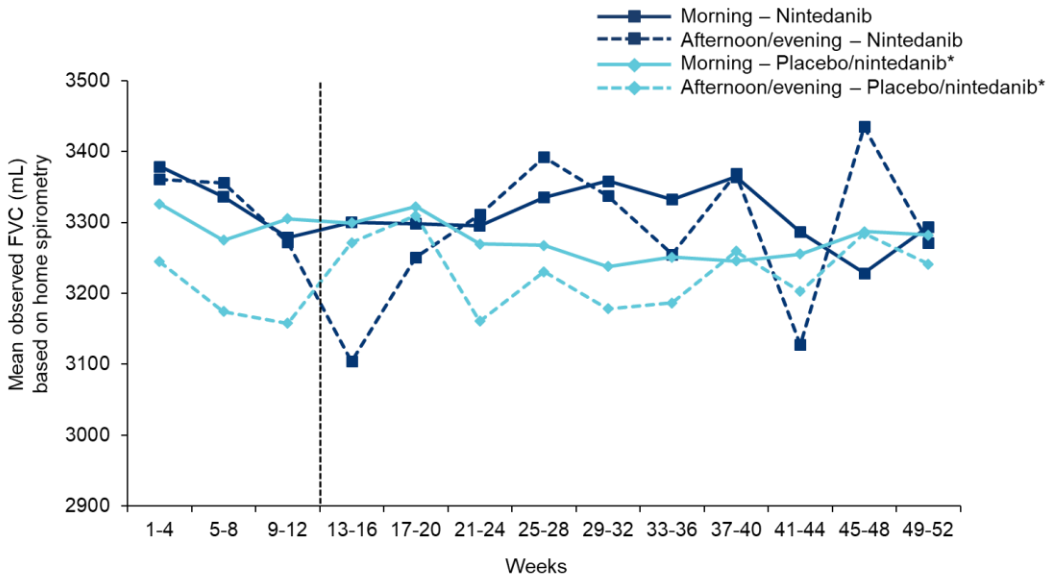

No. of subjects													
Morning – Nintedanib	112	108	109	107	107	105	96	93	91	88	88	87	89
Afternoon/evening – Nintedanib	45	55	51	44	45	47	47	45	50	38	38	47	42
Morning – Placebo/nintedanib*	218	216	208	206	205	197	195	190	187	180	171	170	168
Afternoon/evening – Placebo/nintedanib*	84	84	91	84	88	85	82	73	70	70	81	76	69

*Subjects received placebo (blinded) for 12 weeks followed by open-label nintedanib for 40 weeks.
Morning measurements were taken between 05:00 and 12:00 (inclusive). Afternoon/evening measurements were taken after 12:00 and before 05:00.

Figure S5. Clinic- and home-measured FVC (mL) over time in subjects initially randomised to nintedanib (a) or placebo (b–g) who had an acute exacerbation

a)

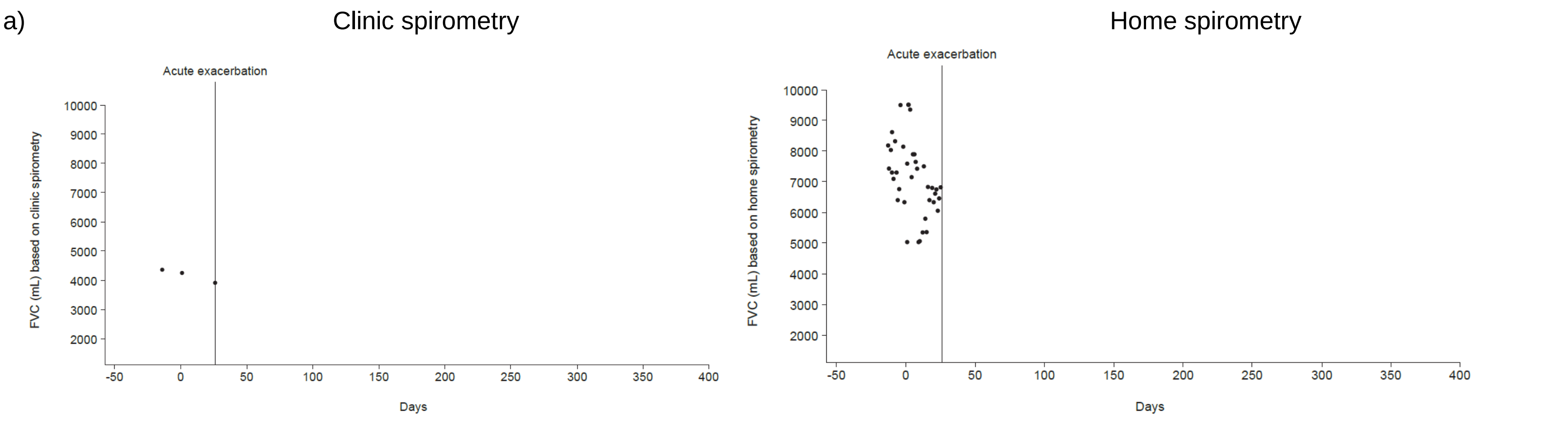

b)

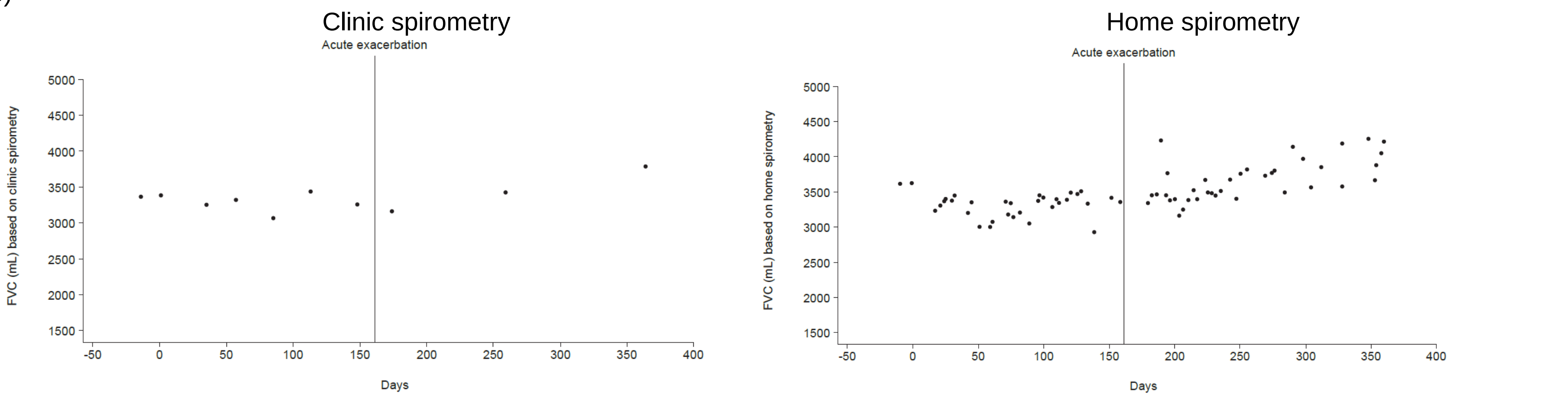

c)

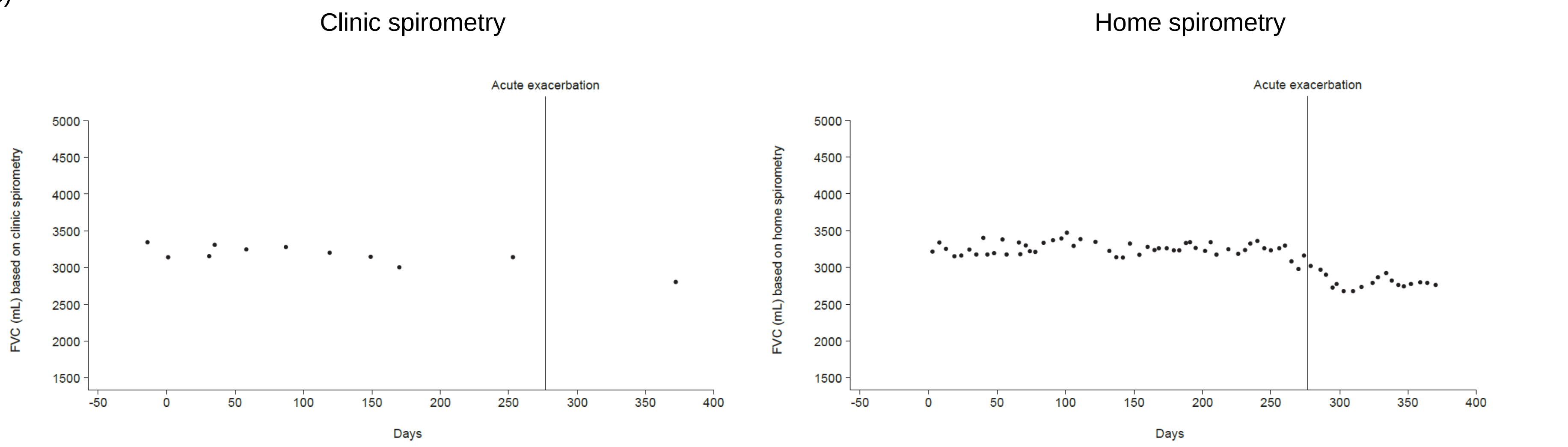
Clinic spirometry
Acute exacerbation
FVC (mL) based on clinic spirometry
Days
Home spirometry
Acute exacerbation
FVC (mL) based on home spirometry
Days

d)

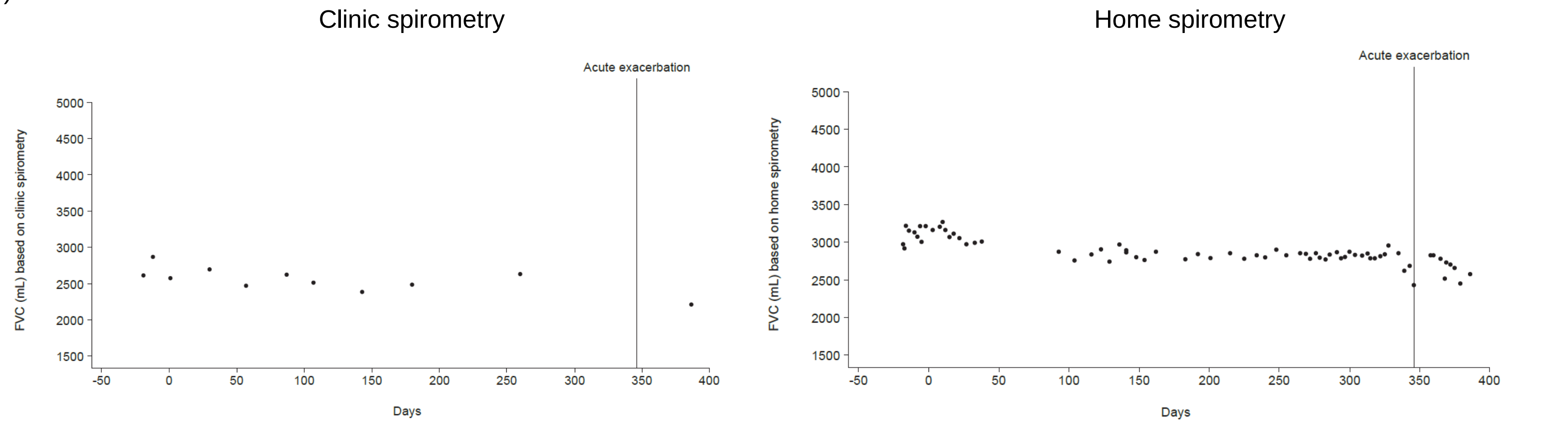
Clinic spirometry
Acute exacerbation
FVC (mL) based on clinic spirometry
Days
Home spirometry
Acute exacerbation
FVC (mL) based on home spirometry
Days

e)

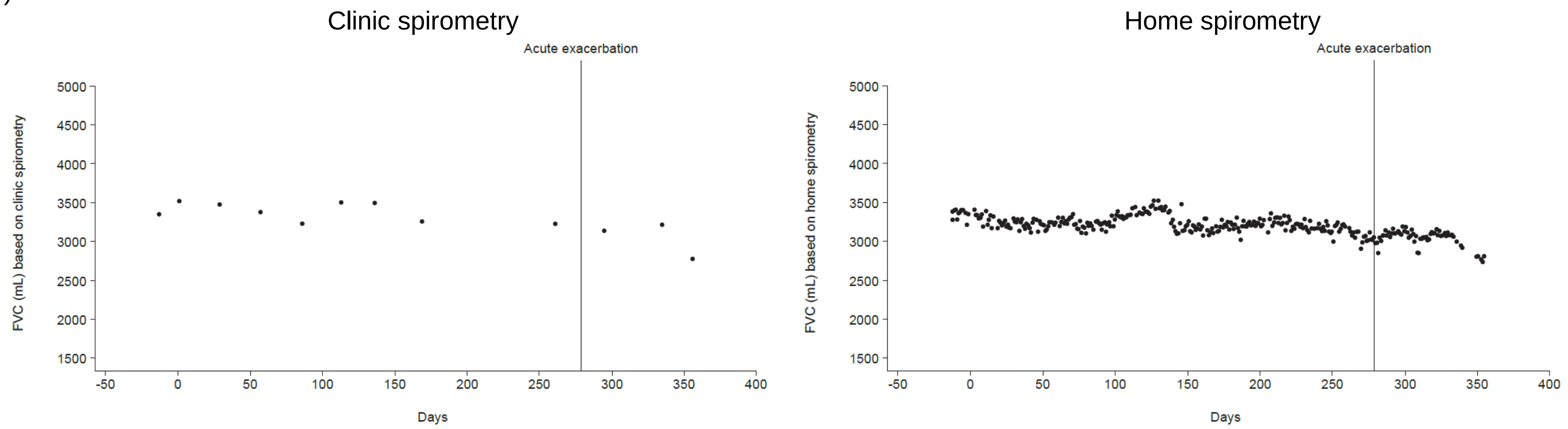
Clinic spirometry
Acute exacerbation
FVC (mL) based on clinic spirometry
Days
Home spirometry
Acute exacerbation
FVC (mL) based on home spirometry
Days

f)

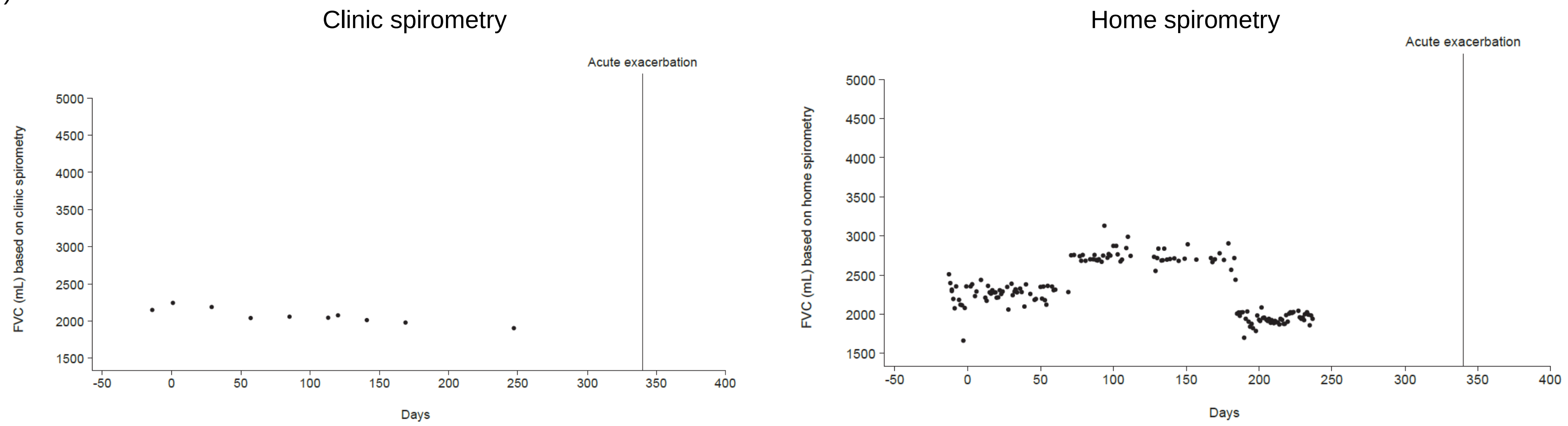
Clinic spirometry
Acute exacerbation
FVC (mL) based on clinic spirometry
Days
Home spirometry
Acute exacerbation
FVC (mL) based on home spirometry
Days

g)

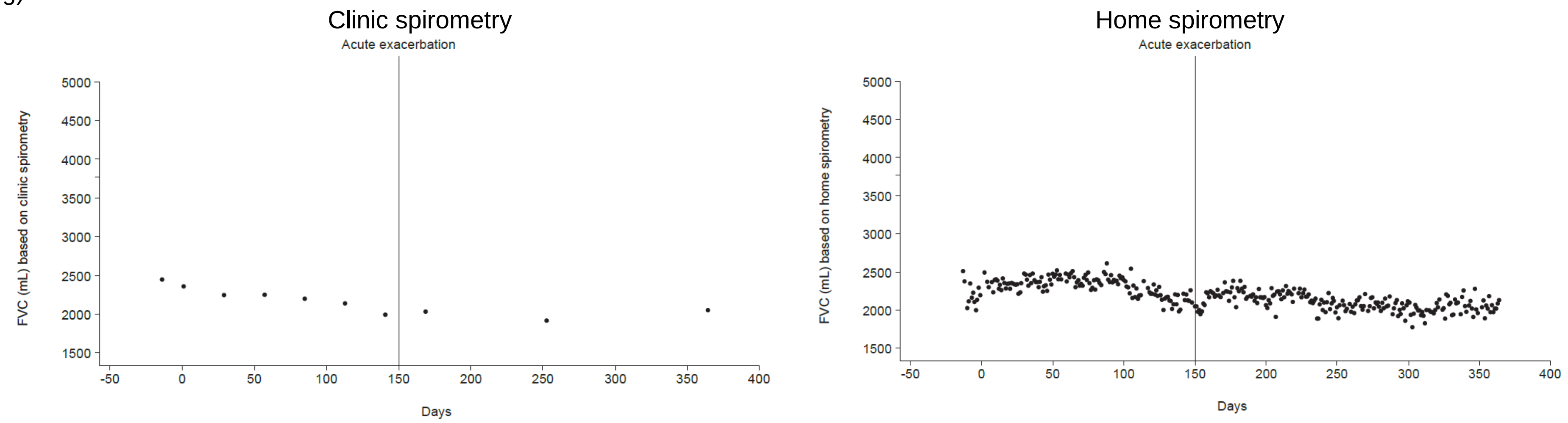
Clinic spirometry
Acute exacerbation
FVC (mL) based on clinic spirometry
5000
4500
4000
3500
3000
2500
2000
1500
-50
0
50
100
150
200
250
300
350
400
Days
Home spirometry
Acute exacerbation
FVC (mL) based on home spirometry
5000
4500
4000
3500
3000
2500
2000
1500
-50
0
50
100
150
200
250
300
350
400
Days

h)

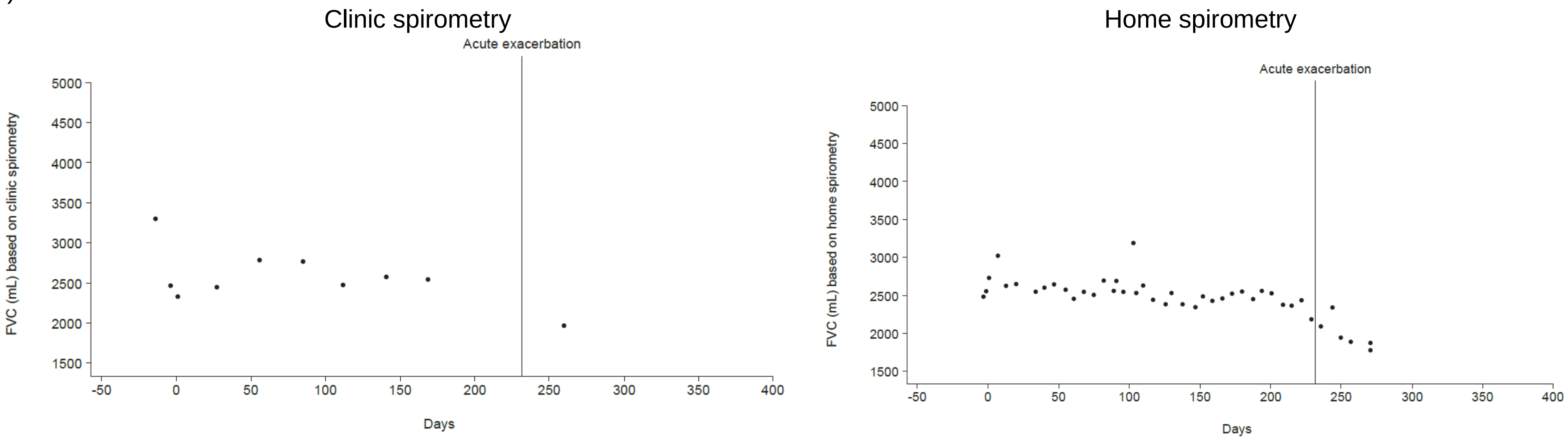
Clinic spirometry
Acute exacerbation
FVC (mL) based on clinic spirometry
5000
4500
4000
3500
3000
2500
2000
1500
-50
0
50
100
150
200
250
300
350
400
Days
Home spirometry
Acute exacerbation
FVC (mL) based on home spirometry
5000
4500
4000
3500
3000
2500
2000
1500
-50
0
50
100
150
200
250
300
350
400
Days
