# Supplementary material for: Home spirometry in patients with idiopathic pulmonary fibrosis: data from the INMARK trial
Source: Eur Respir J. 2021 Jul 8;58(1):2001518. doi: 10.1183/13993003.01518-2020 (PMC8264778; doi:10.1183/13993003.01518-2020)
Supplement: Supplementary file 2 [file ERJ-01518-2020.Shareable.pdf]

# Home spirometry in patients with idiopathic pulmonary fibrosis: data from the INMARK trial

SHAREABLE PDF

Imre Noth[1], Vincent Cottin [2], Nazia Chaudhuri[3], Tamera J. Corte[4,5], Kerri A. Johannson [6], Marlies Wijsenbeek[7], Stephane Jouneau [8], Andreas Michael[9], Manuel Quaresma[10], Klaus B. Rohr[10], Anne-Marie Russell [11], Susanne Stowasser[10] and Toby M. Maher[12,13,14] on behalf of the INMARK trial investigators

[1]Division of Pulmonary and Critical Care Medicine, University of Virginia, Charlottesville, VA, USA. [2]National Reference Centre for Rare Pulmonary Diseases, Louis Pradel Hospital, Hospices Civils de Lyon, Claude Bernard University Lyon 1, Lyon, France. [3]North West Interstitial Lung Disease Unit, Manchester University NHS Foundation Trust, Manchester, UK. [4]Royal Prince Alfred Hospital, Camperdown, Australia. [5]University of Sydney, Sydney, Australia. [6]Medicine and Community Health Sciences, University of Calgary, Calgary, AB, Canada. [7]Dept of Respiratory Medicine, Erasmus MC, University Medical Center, Rotterdam, The Netherlands. [8]Hôpital Pontchaillou – CHU de Rennes, IRSET UMR 1085, Université de Rennes 1, Rennes, France. [9]Syneos Health, Farnborough, UK. [10]Boehringer Ingelheim International GmbH, Ingelheim am Rhein, Germany. [11]College of Medicine and Health, University of Exeter, Exeter, UK. [12]National Heart and Lung Institute, Imperial College London, London, UK. [13]National Institute for Health Research Clinical Research Facility, Royal Brompton Hospital, London, UK. [14]Keck School of Medicine, University of Southern California, Los Angeles, CA, USA.

Corresponding author: Toby M. Maher (t.maher@rbht.nhs.uk)

Check for updates

Shareable abstract (@ERSpublications)

**In a 52-week study in 346 subjects with idiopathic pulmonary fibrosis, mean adherence to weekly home spirometry was 86%. Estimates of the rate of decline in forced vital capacity obtained using home and clinic spirometry were poorly correlated.** https://bit.ly/2WjIQ4b

**Cite this article as:** Noth I, Cottin V, Chaudhuri N, *et al*. Home spirometry in patients with idiopathic pulmonary fibrosis: data from the INMARK trial. *Eur Respir J* 2021; 58: 2001518 [DOI: 10.1183/13993003.01518-2020].

This single-page version can be shared freely online.

Copyright ©ERS 2021

This version is distributed under the terms of the Creative Commons Attribution Licence 4.0.

This article has supplementary material available from erj.ersjournals.com

Received: 1 May 2020
Accepted: 9 Dec 2020

## Abstract

***Background*** Data from the INMARK trial were used to investigate the feasibility and validity of home spirometry as a measure of lung function decline in patients with idiopathic pulmonary fibrosis (IPF).

***Methods*** Subjects with IPF and preserved forced vital capacity (FVC) were randomised to receive nintedanib or placebo for 12 weeks followed by open-label nintedanib for 40 weeks. Clinic spirometry was conducted at baseline and weeks 4, 8, 12, 16, 20, 24, 36 and 52. Subjects were asked to perform home spirometry at least once a week and ideally daily. Correlations between home- and clinic-measured FVC and rates of change in FVC were assessed using Pearson correlation coefficients.

***Results*** In total, 346 subjects were treated. Mean adherence to weekly home spirometry decreased over time but remained above 75% in every 4-week period. Over 52 weeks, mean adherence was 86%. Variability in change from baseline in FVC was greater when measured by home rather than clinic spirometry. Strong correlations were observed between home- and clinic-measured FVC at all time-points (r=0.72–0.84), but correlations between home- and clinic-measured rates of change in FVC were weak (r=0.26 for rate of decline in FVC over 52 weeks).

***Conclusion*** Home spirometry was a feasible and valid measure of lung function in patients with IPF and preserved FVC, but estimates of the rate of FVC decline obtained using home spirometry were poorly correlated with those based on clinic spirometry.
